# Real-world data for 21^st^-century medicine: The clinical and translational science awards program perspective

**DOI:** 10.1017/cts.2023.588

**Published:** 2023-09-11

**Authors:** Pablo Cure, Sanae ElShourbagy Ferreira, Joshua P. Fessel, Deborah Ossip, Martin S. Zand, Scott J. Steele, Kenneth Gersing, Christopher M. Hartshorn

**Affiliations:** 1 National Center for Advancing Translational Sciences, National Institutes of Health, Bethesda, MD, USA; 2 Center for Leading Innovation and Collaboration (CLIC), Clinical and Translational Science Program National Coordinating Center, University of Rochester Medical Center, Rochester, NY, USA; 3 Department of Public Health Sciences, University of Rochester Medical Center, Rochester, NY, USA; 4 Department of Medicine, Division of Nephrology, University of Rochester Medical Center, Rochester, NY, USA

## Background

Real-world data (RWD) refers to data related to patient health status and/or the delivery of healthcare routinely collected from a variety of sources. The clinical evidence derived from analysis of RWD regarding the utilization and potential benefits or risks associated with a medical product or intervention is known as real-world evidence (RWE) [1,2]. Real-world data includes a variety of data sources such as electronic health records (EHR), laboratory and imaging, claims/billing, vital records, digital health technologies (DHT), and other modes of remote data collection that can be obtained retrospectively and/or prospectively during the patient’s continuum of care. In addition, other types of data sources such as environmental exposures, pollution levels (indoor and outdoor), geolocation, text messaging, social media, economic measures, and other sources have the potential to “enrich” health-related data for patients and populations. In the last decade, several advances have brought the potential for RWD applications to a new level: an exponential increase in the sheer quantity of data that can be collected from single and multiple sources; data integration and “ingesting” capabilities from multiple Common Data Models (CDMs); new analytical tools and novel methods such as artificial intelligence (AI) and machine learning (ML); and higher capacity to store and maintain information locally and/or centrally within secure cloud environments.

To keep up with evolving types and uses for RWD, the Food and Drug Administration (FDA) has been developing a series of RWD guidance documents [3] and has launched an Advancing Real-World Evidence Program [4]. In addition, the National Institutes of Health (NIH), the National Cancer Institute (NCI), the National Center for Advancing Translational Sciences (NCATS), and the National Library of Medicine have been addressing the need for standardization around RWD collection and use. For example, since 2017, the FDA-led Common Data Models Harmonization and Open Standards for Evidence Generation project [5] has worked to ensure infrastructure is in place to support standardized data generated across the translational spectrum. These CDMs allow for a unified database model to help in the integration of various RWD sources according to the same standard, facilitating the interoperability of the data from multiple data sources for the conversion of RWD to RWE. During the COVID-19 pandemic, these efforts further materialized in the development of the National COVID-19 Cohort Collaborative (N3C) [6], one of the largest repositories of HIPAA-defined limited data sets in the country that, as of March 2023, includes data from over 77 sites in the U.S. and > 7 million COVID + cases and > 11 million controls, “ingesting” data from a variety of CDMs [6]. Through partnerships among many organizations that provide clinical data, and by securely making data accessible to more than 3,000 researchers, N3C has helped to answer critical questions about COVID-19 biology, clinical behavior, and treatment strategies. For example, one of the first RWD-driven characterizations of the post-acute sequelae of COVID-19 (“long COVID”) was accomplished using N3C [7]. N3C offers one model of how to create a large de-identified database through collection, curation, and analysis of multisite data in a single protected data enclave that can facilitate rapid response to a public health emergency. Recently, to further the potential of these types of RWD approaches, NCATS launched the N3C Public Health Answers to Speed Tractable Results to deliver fast, actionable analyses on pressing COVID-19 questions [8]. Efforts such as N3C can continue to model how institutions, government, and other stakeholders can work together in developing large-scale RWD. In addition, combining clinical/health-related data from EHR-based datasets such as N3C with non-clinical data (e.g., sociodemographic, environmental, geolocation, etc.) can provide a more comprehensive picture of the health and well-being of individuals and communities.

Through its Clinical and Translational Science Awards (CTSA) Program, NCATS supports a national network of biomedical research institutions that accelerate the translation of scientific observations into innovative health solutions [9]. Advancing translational science and improving the efficiency and effectiveness of translation requires a coordinated, collaborative effort. Incorporating RWD into translational science requires combining lessons learned, processes, and know-how from experts and from the communities directly impacted by advances in RWD. As the field rapidly evolves and more data from a wide variety of sources are incorporated into clinical and translational science (CTS) research and day-to-day patient care, the challenges and opportunities presented herein (Table 1) represent a possible blueprint for how the CTSA program could make RWD and RWE a reality to help transform 21st-century medicine.

**Table 1.** Challenges and opportunities in the development and implementation of RWD for RWE in the CTSA program

Key RWD Area	Challenges	Opportunities
*Data Infrastructure, Sources, and Quality*	Data quality control Combining datasets from multiple sources High quality datasets identification Data confidentiality, privacy, and security	Develop and disseminate good quality control practices and use cases Develop guidance on data sources and combining datasets Develop and disseminate AI and ML techniques to improve data quality Sharing best practices and develop novel consent platforms and processes for identifiable participant data
*Integration, Harmonization and Analysis*	Data sharing infrastructure and tools Health needs in RWD/RWE Data privacy and security for multisite data Data harmonization	Disseminate lessons learned from N3C on central data sharing and analytical tools (e.g., Enclave) Local and CTSA consortium-wide health needs in RWD/RWE Best practices for data sharing, privacy, and security standards for the CTSA program Data harmonization best practices and use of CDMs
*Diversity, Equity, Inclusion, and Accessibility*	Data representativeness and access to RWD technology sources Unintended consequences of RWD when evaluating large datasets Priorities in RWD/RWE based on patient, community and population needs Measuring impact of RWD in the health of individuals and populations	Develop local and national standards and strategies for diverse representation based on sociodemographic and geographic variables Lessons learned when using AI and ML in large datasets Community Engagement efforts at CTSA hubs to identify population needs in RWD Using RWD to improve trial recruitment and representation of minorities
*Training, Education, and Career Development in RWD/RWE*	Training and education competencies for next generation Training and resources in data science Real-time dissemination Diversity of workforce in RWD	Develop role-based training competencies and materials Data management training for researchers Provide access to RWD and tools locally and consortium wide to scholars/trainees Disseminate best practices and tools in RWD that can assist researchers RWD competencies and career development opportunities accessible to all Develop standards for Team Science in RWD

CDMs = common data models; CTSA = clinical and translational science awards; N3C = national covid cohort collaborative; RWD = real-world data; RWE = real-world evidence.

## Infrastructure, Sources, and Quality

Data infrastructure, sources, quality, and reliability can be highly variable between individual data ecosystems [10–12]. Further, ethical aspects of use of patient-derived data for research and protection of privacy and confidentiality continue to pose challenges [13]. Informed consent, de-identification, data ownership, linkages, and sharing of data are issues inherent to any RWD effort [14]. Methods to store data and control levels of access for researchers and analysts to specific datasets are as important as the data itself. These challenges are further compounded by continuous and rapid advances in data science and adjacent fields. Together, the challenges require data experts and institutions to constantly re-visit their policies and procedures to maintain the highest standards of quality, reliability, privacy, and security of data and their sources. In addition, data governance and provenance are important parts of the data ecosystem to ensure accuracy and quality of the data collected [15].

**Figure 1.**
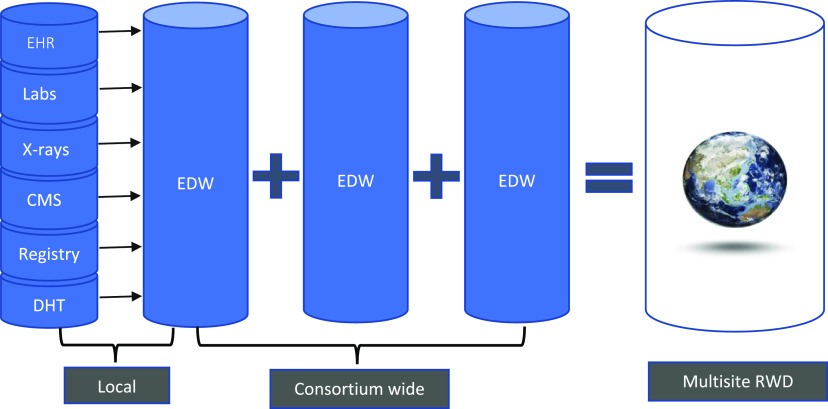
Overall data infrastructure, sources, and integration. CMS = centers for medicare & medicaid services claims data; DHT = digital health technologies; EDW = enterprise data warehouse; HER = electronic health records; Labs = laboratory values; X-ray images; RWD = real-world data.

Capabilities to simultaneously collect/aggregate, securely link, and analyze data from several RWD sources are in great demand to provide a more complete assessment of patients’ and communities’ health (Fig. 1). Currently, EHRs are one of the main data sources for RWD; however, challenges with the quality, reliability, heterogeneity, and utility of the data collected in the EHR continue to be an issue [16–18]. Indeed, EHR systems were developed for clinical documentation, administrative, and billing purposes, so their “re-purposing” as RWD sources for CTS research requires adaptations in data collection, integration, validation, and analysis. Furthermore, overlaying “traditional” sources of RWD with other contextual information can potentially impact data robustness and privacy. Environmental exposures, geolocation, economic measures, and other data extracted from publicly and non-publicly available data sets can enrich analyses. Yet, sources not typically thought of as health-related have different regulations and policies governing their use that must be considered.

Some data sources are well-suited to RWD/RWE applications. For example, disease or specialty-specific registries and databases have successfully supported new indications for existing drugs [19–21]. These registries require strategic planning – incorporating adequate governance, infrastructure, resources, and expertise beyond the registry itself – and flexibility, allowing for dynamic evolution to bring in novel RWD data sources and methods that bolster the registry’s utility. One example is the NCI Surveillance, Epidemiology, and End Results cancer registry [22,23], which provides a high-quality population base (state or metropolitan area) from central cancer registries that enable monitoring of disease prevalence, cancer population needs, and health disparities and their impact in these communities. Bringing RWD together from various sources is only the first step. Generating RWE requires making RWD research-ready and employing adequate and sometimes sophisticated analytical methods.

Data collected from patients and/or populations requires the highest ethical, confidentiality, and privacy standards. Techniques such as de-identification, aggregation, data pooling, and other methods to maintain privacy should be in place both locally (at institutions) and centrally (within a central enclave environment). Further advances in obtaining informed consent, such as e-consent, should be up to speed with the pace of technologies used to collect participant data and their willingness to be contacted for future participation in prospective trials.

## Integration, Harmonization, and Analysis

Combining data from multiple sources and systems requires harmonization of the data being collected to facilitate future evaluations and analyses. As the ability to integrate data from multiple sources improves, evidence from single data sources may become less preferable. However, the process of data harmonization and curation is currently resource-intensive. Harmonizing CDMs of various networks or data sources also allows researchers to ask specific data questions of potentially much larger (combined) sources of RWD. It is tempting to combine as much data as possible, but it is critical to first identify the purpose, timepoints, and desired impact of data and collect it in a way that minimally burdens patients, healthcare providers, and others at the data generation source. Further, it is important to adhere to the highest possible data quality standards. RWD data from multiple sources often improves data richness, but not all data will be equally useable without proper integration and harmonization.

When combining data from multiple data sources, maintaining privacy and security must be a focus of RWD/RWE efforts. Privacy-Preserving Record Linkage (PPRL) is one way of connecting records that refer to the same individual across different data sources using secure, pseudonymization processes while maintaining the individual’s privacy [24]. NCATS is piloting PPRL technology in the N3C to determine if linking multiple data sets enhances usability of COVID-19 RWD. Identifying high-quality data and mapping their provenance allows for selection of the best and most representative data for multisite analyses.

Despite rapid advances, there are important remaining challenges for broader use of multimodal RWD. For example, errors – inherent to each source and/or arising during harmonization – could confound the process of RWD integration and must be addressed. More robust interoperability for CDMs and codes of conduct to reduce misuse of research findings from RWD and research data in general is necessary. Additionally, newly formed federated or centrally kept data environments must incorporate tools to enable use of completely de-identified or HIPAA-limited data for additional flexibility and adaptation to both institutional and multisite data aggregation needs. Efficient and meaningful integration of data from multiple sources and institutions could help create the next generation of data systems for evidence-based medicine and real-time clinical support [25–27].

RWD can also come from more specific – disease-based – fully consented registries which include strict policies and procedures for data linkages and patient-identified data requests in order to protect patient privacy [28]. In these cases, data harmonization is mostly unnecessary with all data points and variables specified from the outset allowing for more specific questions and outcomes of interest (such as mortality, disease burden, laboratory results, and other variables) to be followed over time. However, as with well-designed clinical trials, research with registries must include *a priori* definition of meaningful outcomes to evaluate and pre-defined analytical methods to prevent or diminish bias.

Novel analytical techniques utilizing AI and ML can provide additional capacity to help analyze large amounts of data and identify “subtle” risk factors that may not be easily identified through more conventional analysis. At the same time, utilizing both automated and manual data review techniques can be used for data quality control and assurance in large multisite data efforts to improve RWD obtained locally and in aggregate with automatically maintained data provenance as a requirement [29].

## Diversity, Equity, Inclusion, and Accessibility (DEIA)

Insufficient diversity, equity, inclusion, and accessibility (DEIA) considerations in planning, analyzing, and collecting data can introduce bias and limit the ability of RWD to develop meaningful RWE. Gichoya et al., developed an AI algorithm that accurately identified self-reported race from imaging data only, a phenomenon that could not be replicated by human radiologists and that was not readily explainable [30]. This deliberate example highlights the fact that AI algorithms can make race-specific conclusions based on factors invisible to human evaluators. If those cryptic conclusions are erroneous, or worse, perpetuate biases, they could lead to healthcare decisions that perpetuate health disparities. Of course, these kinds of models may also accelerate beneficial discoveries that would be otherwise difficult to achieve. To ensure the most beneficial patient and public health outcomes, the potential for introduction and amplification of biases or structural inequities must be reckoned with proactively.

Equitable RWE begins with high-quality, representative RWD. Addressing the “digital divide” and fair access to DHTs is also central to discussions of DEIA in RWD. Technologies are becoming more widely used to assess health parameters – e.g., continuous glucose levels, vital signs, and physical activity. Such strategies can adapt to users across the lifespan and expand testing of interventions or therapeutics to more diverse or rural communities or even monitor activity and health in space [31]. Low (e.g. text messaging)- and high-tech (e.g. smart apps) options need to be available and tailored to users to promote equitable access to and representativeness of RWD for all populations intended to benefit.

To advance DHTs, other DEIA-related roadblocks and access questions must be addressed. Services and support tools built and validated to promote inclusivity and equity in data collection and analytical technologies are crucial. Building trust in the use of novel technologies for the benefit of patients and communities is part of the providers’ role in the development and implementation process. At the same time, patient/user input in the development and optimization of new technologies plays an important role in improving utilization, user satisfaction, and adherence. The million-dollar question and challenge for CTS researchers remains: how do we align 21st-century RWD with 21st-century medicine for the benefit of all? The answer requires the enterprise to focus on integrated approaches intentionally attuned to DEIA considerations.

## Training, Education, and Career Development in RWD/RWE

Training the next generation of scientists is one of the main goals of NIH. For example, the CTSA program supports trainees and scholars through a number of funding opportunities such as the KL2 program (now K12), TL1 program (now T32 pre-doctoral and T32 post-doctoral), as well as other programs such as diversity, reentry, and reintegration supplements [32]. Each program is geared towards a specific phase in the training and career development of the trainee/scholar. Identifying training opportunities, activities and resources tailored to the specific trainee/scholar needs in data management, analytics, and reporting can be crucial in the development of a highly skilled/data-driven workforce of the future. Recently, the CTSA program diversity was published showing areas where we can improve to attract a more diverse workforce in clinical and translational science [33]. Specialized pieces of training in data management and novel analytical methods using AI and ML can also help in the development and retention of professionals focused on health and related RWD in this fast-growing field. Facilitating an environment where cross-communication between clinicians, data managers, data analysis experts, regulatory authorities, and the community can provide the catalyzing force to maximize efficiency, impact, and return on investment of RWD-oriented projects. As in the device development “world” where clinicians, bioengineers, technology transfer, and marketing experts come together to solve unmet medical needs, we need to develop a similar team science approach where access to specialized resources and expertise in RWD can bring data managers, experts in data analysis (including AI and ML data experts) and others, closer to their clinical/scientific counterparts to help answer meaningful health questions. All needs to be done in parallel with identifying pressing community and population health needs and questions that if answered through RWD could help to significantly improve individual and public health locally and nationally.

## Discussion

**Figure 2.**
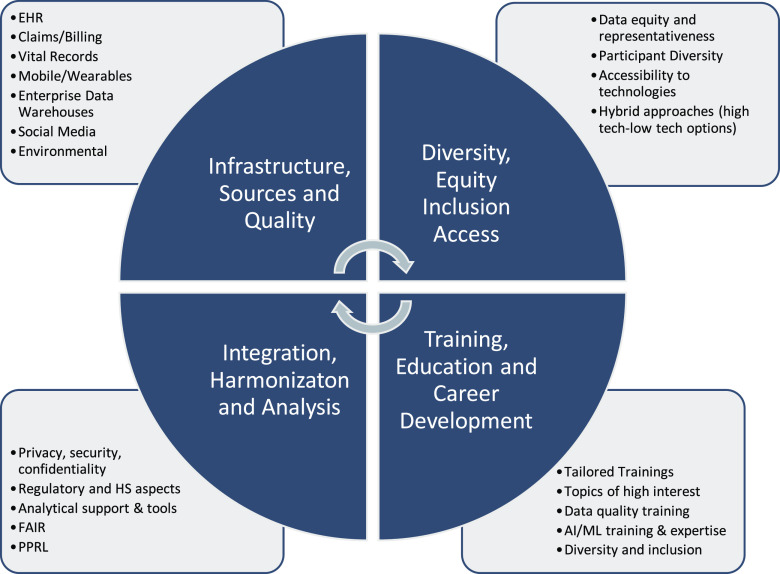
Key areas in real-world data within CTSA programs. D&I = dissemination and implementation; FAIR = findable, accessible, interoperable, reproducible; HER = electronic health records; HS = human subjects; PPRL = privacy-preserving record linkages.

To make RWD and RWE a reality within the CTSA Program, investigators, research participants, clinicians, patient advocates, funding agencies, regulatory agencies, industry, and many others need to collaboratively identify strategic priorities that maximize impact of data on scientific knowledge and health outcomes. Developing generalized, intervention-agnostic approaches driven by translational science could be an area for consideration by the CTSA consortium. Several key areas already align with CTSA infrastructure and resources (Fig. 2).

Furthermore overlaying “traditional” sources of RWD with other information that can potentially impact health and health outcomes, such as environmental exposures, geolocation, place of residence, economic measures, and other data extracted from publicly and non-publicly available data sets (e.g., from social networking sites such as Facebook, LinkedIn, Instagram, etc., or even data from the “Internet of Things”) can add richness (and further complexity) to the data. Yet, these datasets derive from sources not typically thought of as being health-related and that may have different regulations and policies governing their use could create additional challenges in the successfully translating RWD into RWE. Integration of RWD from smaller, well-designed, fit-for-use disease registries as well as other prospective hypothesis-generating types of observational datasets requires *a priori* definition of meaningful outcomes to evaluate using pre-defined analytical methods to minimize bias.

Several limitations must be taken into consideration when utilizing electronic health records data from efforts such as N3C, including overrepresentation of certain populations such as patients with more access to health services, high utilizers of health care, patients with more severe symptoms, and conditions and inpatients. In addition, clinical follow-ups outside of the health system, as it happens in community hospitals or other outside-of-the-health-system settings (e.g. private doctors’ offices) can also be missing/not recorded and therefore outcomes are limited to data within the enclave [7]. It is therefore important to acknowledge the limitations of these large RWD sources before making broad population-based conclusions. Data privacy, security, and consent for future contact remain cornerstones to make sure the research performed using RWD through large and broad datasets or smaller and more controlled datasets (e.g., registries) can have all the necessary safeguards to allow for an ethical and scientifically rigorous process.

Federated or centralized approaches both provide advantages and disadvantages when it comes to data sharing, integration, harmonization, and quality [29]. For example, efforts to improve data quality in N3C and providing those data to institutions are now part of the feedback received by institutions sharing data in the N3C data enclave. Using federated and centralized approaches to collect RWD can also help with both data quality, by providing feedback to institutions contributing data in a centralized platform/data enclave, or by performing participant screening for future trials in a federated environment, under the right participant consent for future contact. Data standardization at the collection source also represents a major deficiency of large, integrated datasets. Establishing standards for data entry at the outset (during the clinical encounter or shortly after) as well as quality control methods while maintaining data provenance remains critical.

Training the next generation of data managers, statisticians, clinical informaticians, data clinicians and other experts within the CTSA collaborative/team science approach can greatly enhance the capability of the consortium to timely respond to current and future public health needs using RWD. Well-developed, customizable, complementary, and competency-based training programs may represent one of the biggest opportunities for developing the field of RWD and data science within the CTSA consortium. In addition, basic principles of data management and novel methods training could bring great added value to current and future clinical researchers.

Combining these CTSA assets and developing innovative approaches to improve the quality, utilization, and reproducibility of RWD findings under FAIR (Findable, Accessible, Interoperable, and Reproducible) guiding principles [34], can bring new and critical scientifically sound programmatic activities to fruition. Designing approaches to validate and implement new technologies and analytical tools can accelerate RWE advances. Issues of DEIA, including equitable access to technologies and representativeness of data, need to be addressed from the start and not as an afterthought. Further, applying an equity lens to securely source, integrate, and harmonize clinically relevant, high quality, representative RWD could result in RWE-based approaches that transform healthcare and enhance patient health – as the ultimate goal of all RWE is to answer real-world questions and deliver real-world returns, to all.
